# Device-Based Measures of Sedentary Time and Physical Activity Are Associated With Physical Fitness and Body Fat Content

**DOI:** 10.3389/fspor.2020.587789

**Published:** 2020-12-18

**Authors:** Jani P. Vaara, Tommi Vasankari, Thomas Wyss, Kai Pihlainen, Tommi Ojanen, Jani Raitanen, Henri Vähä-Ypyä, Heikki Kyröläinen

**Affiliations:** ^1^Department of Leadership and Military Pedagogy, National Defence University, Helsinki, Finland; ^2^The UKK Institute for Health Promotion Research, Tampere, Finland; ^3^Swiss Federal Institute of Sport Magglingen, Magglingen, Switzerland; ^4^Personnel Division of Defence Command, Helsinki, Finland; ^5^Finnish Defence Research Agency, Finnish Defence Forces, Helsinki, Finland; ^6^Faculty of Social Sciences (Health Sciences), Tampere University, Tampere, Finland; ^7^Faculty of Sport and Health Sciences, University of Jyväskylä, Jyväskylä, Finland

**Keywords:** cardiorespiratory fitness, muscular fitness, objective physical activity, sedentary time, BMI-body mass index, waist circumference

## Abstract

**Introduction/Purpose:** Physical activity and sedentary time may associate with physical fitness and body composition. Yet, there exists some observational studies that have investigated the associations of device-based measures of sedentary time and physical activity (PA) with cardiorespiratory fitness (CRF) and body composition but associations with muscular fitness (MF) are less studied.

**Methods:** Objective sedentary time and physical activity was measured by a hip worn accelerometer from 415 young adult men (age: mean 26, standard deviation 7 years). Cardiorespiratory fitness (VO_2_max) (CRF) was determined using a graded cycle ergometer test until exhaustion. Maximal force of lower extremities was measured isometrically and lower body power was assessed using standing long jump (MF). Body composition was determined with bioimpedance method. Single and compositional approach was used in regression analysis.

**Results:** Mean sedentary time was 707 (standard deviation 133) minutes per day (77 ± 8% of the wear time). Volumes of all PA intensities were positively associated with CRF and associations showed linearly increasing magnitudes with higher intensities in single regression models adjusted for age and smoking (*p* < 0.001). Similarly, PA intensities were positively associated with lower body MF, however, with weaker associations (*p* < 0.005). After further adjustment for resistance training, the associations remained significant. The associations of the relative distribution of time within sedentary behavior (SB), light intensity PA (LPA) and moderate-to-vigorous PA (MVPA) behaviors as a whole with using compositional analysis further revealed that within the composition MVPA and SB were positively associated with CRF and MF (*p* < 0.001), while LPA was not. In addition, within the composition, accumulated PA bouts lasting more than 3 min were consistently associated with CRF and MF, and with all body composition variables (*p* < 0.001), while sedentary time was associated with body fat percentage (*p* < 0.001).

**Conclusion:** Promoting physical activity and reducing sedentary time may have positive influence on physical fitness and body fat content, and thereby may offer positive health effects. Physical activity of higher intensities may offer greater benefits.

## Introduction

Physical fitness has been recognized as an important determinant of health-related outcomes and can be divided to cardiorespiratory and muscular fitness. Cardiorespiratory fitness refers to body‘s capacity to uptake oxygen for energy production use in cells, whereas muscular fitness refers to capacity of a muscle or muscle group to generate force either maximally or repeatedly within a given time. Numerous studies have shown cardiorespiratory fitness to be a significant predictor of health-related issues (Kodama et al., 2009; Ross et al., 2016) and although mostly with smaller magnitude, muscular fitness has been shown to associate with health outcomes independent of cardiorespiratory fitness (Vaara et al., 2014; Grøntved et al., 2015). Although, physical fitness is partly inherited capability it is modifiable via health behavior including physical activity (PA) and sedentary behavior (Booth et al., 2012).

Observational studies have demonstrated that self-reported physical activity is associated with cardiorespiratory fitness (e.g., Emaus et al., 2010; Porter et al., 2017). Interestingly, there is some evidence that also self-reported sedentary time could be related to cardiorespiratory fitness. Eriksen et al. (2016) observed that self-reported sedentary time was inversely associated with cardiorespiratory fitness in those individuals who engaged only little physical activity, whereas in moderately or highly physically active individuals there was no similar association. The latter finding may therefore, emphasize that for sedentary individuals even replacing some of the sedentary time with physical activity could lead to improvement of cardiorespiratory fitness and thereby mediate health benefits, although conflicting results have also been observed (Barlow et al., 2016; Porter et al., 2017). Nevertheless, self-report methods are known to be concerned and limited to bias (Hukkanen et al., 2018), such as over-reporting. For example, a study by Tucker et al. (2011) observed that self-reported moderate physical activity was over reported by more than 4 h and vigorous physical activity by 26 min per week in US adults. Therefore, device-based physical activity monitoring could offer more precise measures of volume of physical activity within different intensity thresholds objectively and also measure sedentary time.

Objectively measured physical activity has been observed to associate with cardiorespiratory fitness in adults (Nokes, 2009; Cao et al., 2010a,b; Kulinski et al., 2014; Dyrstad et al., 2016) and children (Gralla et al., 2016; Collings et al., 2017; Joensuu et al., 2018). Kulinski et al. (2014) reported in adults that objectively measured sedentary time was inversely associated with cardiorespiratory fitness independent of physical activity. Similarly, an inverse association between objectively measured sedentary time and cardiorespiratory fitness has been shown in older individuals (Jantunen et al., 2017). Although the relationship between physical activity and cardiorespiratory fitness is well-evidenced, there are, however, considerably less studies of objective physical activity and muscular fitness components, especially in adults. Joensuu et al. (2018) reported an inverse association between muscular fitness and objective physical activity, but not with sedentary time in children, and a recent review concluded positive association of objectively measured MVPA and vigorous physical activity, but not sedentary time, with strength and power in children (Smith et al., 2019).

In addition to physical fitness, also body composition is related to physical activity. Inconsistent findings of the relationships between self-reported physical activity with adiposity have previously been demonstrated in observational studies in adults (Fogelholm and Kukkonen-Harjula, 2000; Wilks et al., 2011; da Silva et al., 2019). The controversial findings may, to some extent, exist, because of more commonly used self-report methodology than objective measures of physical activity. Recent studies have shown that the association between objectively measured physical activity and adiposity markers are stronger than those of self-report measures (Wanner et al., 2017; Guo et al., 2019). Associations of device based measures of physical activity and sedentary time with adiposity markers have been observed in adults (Johansson et al., 2019, 2020; Lee et al., 2019) and in children (Stevens et al., 2007; Sögüt et al., 2019) Therefore, there is a need for studies assessing objective physical activity including sedentary time and physical activity intensities and investigate their associations with adiposity outcomes. The purpose of the present study was to explore the associations of objectively measured physical activity and sedentary time with physical fitness including both cardiorespiratory and lower body muscular fitness, and with adiposity outcomes in a population-based sample of young Finnish adult men.

## Methods

### Participants

Participants consisted of 776 young (age: mean 26, standard deviation 7 years) adult Finnish men, who were invited in the military refresher training. The information about the study plan for participants were sent to the participants 5 months before the study conduction, which were carried out in 7 different measurement sessions during 2015 (May-November). Altogether, 1,106 men were called up to military refresher training and 823 participated in the training lasting 3–5 days. The most typical reasons for non-participation to the military refresher training were related to personal reasons, such as work, study-, or health-related issues. Out of the 823 men, 776 men volunteered to participate in the study. Of the 776 men, 519 were randomly selected to the follow-up measures of accelerometer physical activity. The final study sample consisted of 415 participants meeting the inclusion criteria of a minimum of 4 days with at least 10 h accelerometer wear time per day. The study protocol was explained in detail to the participants before they gave their written consent. The study was approved by the ethical committees of the University of Jyväskylä and the Central Finland Health Care District, as well as the Defence Command of the Finnish Defence Forces (AM5527). The whole study sample was compared with corresponding cohorts of 20 to 30 years old Finnish men in the national register data (Statistics Finland) from the year 2014 for education and place of residence. Based on these analyses, in the current study sample Northern and Southern Finland were slightly over-represented. In addition, the proportion of those participants who had studied 13 years or more was slightly over-represented. Taken into account previous limitations, the whole study sample can be considered to represent young adult Finnish men.

### Objectively Measured Physical Activity and Sedentary Time

A waist-worn triaxial accelerometer (Hookie AM 20, Traxmeet Ltd., Espoo, Finland) was used to quantify physical activity and sedentary time. The accelerometer was attached to the right side of the hip with a flexible belt. Participants were given instructions to wear the accelerometer for 7 consecutive days during wake time. The acceleration data were collected at 100 Hz sampling rate and the raw accelerometer data were stored on a hard disk for further analysis. The mean amplitude deviation (MAD) values of the resultant acceleration of the three orthogonal acceleration components were determined in 6 s epochs. The MAD values have been found to be a valid indicator of incident energy consumption during locomotion (Vähä-Ypyä et al., 2015a,b) The MAD values were converted to METs for each epoch.

Physical activity was stratified into three intensity categories regarding METs: light physical activity (LPA) 1.5–2.9 MET; moderate physical activity (MPA) 3.0–5.9 MET and vigorous physical activity (VPA) >6.0 MET (Husu et al., 2016) and also total physical activity was calculated as the combined amount of light, moderate, and vigorous activities. Sedentary time was defined as the time spent in the lying, sitting, and standing positions without movement (<1.5 MET) (Vähä-Ypyä et al., 2018). In addition, the epoch-wise MAD values were converted to METs and 1, 10, and 30 min exponential moving average of the MET values were calculated for each epoch. The maximum peak MET levels of weekly exponential moving average values (e.g., 1, 10, and 20 min) were identified (Vasankari et al., 2017).

*Cardiorespiratory fitness* (VO_2_max) was determined using an indirect graded cycle ergometer test (Ergoline 800S, Ergoselect 100K, Ergoselect 200K, Bitz, Germany) until exhaustion. A progressive protocol was used, which initially started at a power output of 50 W and was increased 25 W every 2 min until exhaustion. Heart rate (HR) was continuously recorded during the test (Polar Vantage NV or S610, S710, or S810, Kempele, Finland). Predicted VO_2_max was estimated from HR and maximal power (W) (Fitware, Mikkeli, Finland) with the following equation: VO_2_max (ml·kg^−1^·min^−1^) = 12.35 × Pmax/kg + 3.5, where Pmax is maximal power and kg is body mass in kilograms. The intra-class correlation has been reported to be high with this method (Santtila et al., 2013).

*Muscular fitness tests* consisted of push-ups, sit-ups, standing long jump and maximal isometric force of lower and upper extremities. In the present study, standing long jump and maximal isometric force of lower extremities were only used for further analysis because objectively measured physical activity measures movement conducted by the lower limbs of the body. Correct technique was demonstrated to participants before each test and only the trials with adequate technique were accepted. The warm-up prior the tests lasted 10 min and consisted of calisthenics exercises, such as x-jumps, push-ups, sit-ups, squats, planks, and countermovement jumps. Standing long jump test was performed in the specifically designed gym mat (Fysioline, Tampere, Finland). The participants were instructed of the correct technique prior the testing, and each of them performed several jumps after a warm-up and prior the testing. The participants were instructed to jump (horizontally) forward as far as possible from a standing position, using a prior countermovement and hands freely swinging by their sides without falling backward upon bilateral landing. The participants completed 3 trials each interspersed by a 1-min rest period. The performance was measured with 1 cm precision. Maximal isometric force was measured with horizontal leg press (a test for maximal strength) using a dynamometer. Knee angle was set to 107° with a goniometer, and hands were placed on a handle grip in a leg extension test. A warm-up series of at least 2 submaximal sets were done prior to maximal sets. Three trials were performed using a 30-s recovery period. The best performance was included in the analysis. Each participant was advised to produce maximal force as fast as possible and to maintain it for 3 s. The participants were verbally encouraged during the maximal efforts by the test personnel. Lower body muscular fitness index was calculated firstly converting both standing long jump and isometric force of lower extremities to z-scores. Thereafter, these z-scores were summed together and then standardized as a final composition of lower body muscular fitness z-score.

### Body Composition

Body composition and body weight was determined with bioelectrical impedance method (Inbody 720, Biospace Company, Seoul, Korea). Body weight was measured to the nearest 0.1 kg and body height by a commercial scale to the nearest 0.1 cm. Body mass index (BMI) was calculated and waist circumference was measured at the level of iliac crest after exhaling by a tape measure.

### Demographic and Background Variables

Age and smoking habits (smoking, non-smoking, quit smoking) were assessed with a questionnaire. Self-reported resistance training was also assessed with a questionnaire and was used as a covariate in the statistical analysis (resistance training 0–2 times a week vs. > 2 times a week).

### Statistics

Data was analyzed with IBM SPSS Statistics software (SPSS for Windows 25.0.0.1) and R-software. Descriptive statistics as means, standard deviations, and 95% confidence intervals (CI) were calculated. A similar approach was used as by Chastin et al. (2015), where firstly, linear regression models and secondly, compositional analysis were used (CoDaPack-software, v2.03.11). This approach takes into account that time is finite during the day and thus time spent in different behaviors are codependent. Linear regressions were used to calculate standardized regression coefficients (β) with 95% CIs *p*-level set at <0.05 with using age and smoking as covariates. In order to properly consider the relationship between different sedentary and physical activity behaviors, we further used a compositional data approach (Chastin et al., 2015). First we re-scaled the original time variables in different behaviors up to 1 (i.e., they varied between 0 and 1 and the sum of them was 1) and secondly, we made an isometric log-ratio (ILR) transformation for these proportions (Chastin et al., 2015). Finally, the new ILR-transformed variables were used as exposure variables in linear regression models adjusted for age and smoking. Moreover, analysis of variance was used with Tukey HSD *post-hoc* test for normally distributed variables and for not normally distributed variable Kruskall-Wallis test was used with Wilcoxon *post-hoc* test. In addition, the physical fitness variables were stratified to low (lowest 20%), moderate (20–80%), and high (highest 20%) subgroups in cardiorespiratory and lower body muscular fitness variables. Moreover, the associations of sedentary time and PA intensities across the subgroups were studied using analysis of covariance with age and smoking as covariates (Table 4). Similarly, established cut points for BMI and waist circumference were used with analysis of covariance with age and smoking as covariates (Table 7). To study the joint associations of low and high moderate-to-vigorous physical activity with low and high sedentary time, the study sample was divided into low group (lowest 20%) and high group (highest 20%) (Table 8). There were no individuals with low sedentary time combined with low MVPA and no individuals with high sedentary time combined with high MVPA. Thus, the differences between combined low sedentary time and high MVPA were analyzed against high sedentary time and low MVPA group in cardiorespiratory fitness, lower body muscular fitness, body mass index as well as waist circumference with paired-samples *t*-test (Table 8).

## Results

The demographic and baseline characteristics are presented in the Table 1. Participants‘ mean sedentary time was 707 (standard deviation (SD) 133) minutes per day, which consisted of 77% (sd 8) of their wear time. Participants spent on average 122 (sd 47) min (13.5 ± 5.2% of the wear time) in LPA, 79 (sd 33) min (8.7 ± 3.6% of the wear time) in MPA and 4.4 (sd 6.2) min (0.5 ± 0.7% of the wear time) in VPA.

**Table 1:** Demographic and baseline characteristics in the study population (*n* = 415).

**Characteristics**	**Mean (standard deviation)**	**95% confidence interval**
Age in years	28.5 (7.3)	27.8–29.2
Smokers (%)	23.2	
**Sedentary time**		
Total sedentary time (min)	706.9 (132.6)	694.1–719.7
Total sedentary time (relative to wear time, %)	77.3 (8.1)	76.6–78.1
**Mean daily accumulated sedentary** ** time (min) from bouts lasting**		
<3 min	215.6 (63.9)	209.4–221.7
3–10 min	175.2 (48.3)	170.5–179.9
11–19 min	141.3 (51.5)	136.3–146.2
20–60 min	153.2 (87.7)	144.8–161.7
>60 min	21.4 (29.9)	18.5–24.3
**Mean daily accumulated sedentary time** ** relative to wear time (%) from bouts lasting**		
<3 min (%)	23.8 (7.0)	23.2–24.5
3–10 min (%)	19.3 (4.9)	18.8–19.7
11–19 min (%)	15.4 (5.0)	14.9–15.9
20–60 min (%)	16.5 (8.5)	15.7–17.3
>60 min (%)	2.3 (3.1)	2.0–2.6
**Physical activity**		
Light activity (1.6–3 MET) (min)	121.9 (47.3)	117.4–126.5
Moderate activity (3–6 MET) (min)	78.6 (32.5)	75.5–81.8
Vigorous activity (>6 MET) (min)	4.4 (6.2)	3.8–5.0
Moderate-vigorous activity (≥3 MET) (min)	83.0 (33.9)	79.8–86.3
Total activity (≥1.5 MET) (min)	205.0 (73.4)	197.9–212.1
Light activity (1.6–3 MET) (%)	13.5 (5.2)	13.0–14.0
Moderate activity (3–6 MET) (%)	8.7 (3.6)	8.4–9.1
Vigorous activity (>6 MET) (%)	0.5 (0.7)	0.4–0.5
Moderate-vigorous activity (≥3 MET) (min)	9.2 (3.8)	8.8–9.6
Total activity (≥1.5 MET) (min)	22.7 (8.1)	21.9–23.4
**Mean daily accumulated physical activity (>1.5 MET) (min) from bouts lasting**		
<3 min	170.6 (63.7)	164.4–176.7
3–10 min	24.7 (17.3)	23.0–26.4
11–19 min	6.3 (7.4)	5.6–7.0
20–60 min	3.0 (6.5)	2.3–3.6
>60 min	0.4 (2.5)	0.2–0.7
**Mean daily accumulated physical activity (>1.5 MET) relative to wear time (%) from bouts lasting**		
<3 min (%)	18.9 (7.0)	18.2–19.5
3–10 min (%)	2.7 (1.9)	2.5–2.9
11–19 min (%)	0.7 (0.8)	0.6–0.8
20–60 min (%)	0.3 (0.7)	0.3–0.4
>60 min (%)	0.05 (0.30)	0.02–0.07
**METs, maximum peak MET-level of weekly** ** physical activity bouts lasting**		
1 min	6.5 (1.6)	6.4–6.7
10 min	4.2 (1.1)	4.0–4.3
30 min	3.2 (0.9)	3.2–3.3
**Physical fitness and Body composition**		
Cardiorespiratory fitness (ml/kg/min)	41.3 (8.0)	40.6–42.1
Sit-ups (reps/min)	35 (12)	34–36
Push-ups (reps/min)	29 (14)	28–31
Standing long jump (cm)	226 (26)	224–229
Maximal isometric force of the lower extremities (kg)	343 (90)	334–352
Maximal isometric force of the upper extremities (kg)	88 (21)	86–90
Waist circumference (cm)	88.3 (11.1)	87.2–89.4
Body mass index	25.5 (4.1)	25.1 ± 25.9
Body fat%	17.7 (7.9)	17.0–18.5
Body mass (kg)	82.1 (15.2)	80.7–83.6
Body height (cm)	179.4 (6.3)	178.8–180.0

### Associations of Sedentary Time and Time Spent in Different Physical Activity Intensities With VO_2max_ and Muscular Fitness

Table 2 demonstrates that both sedentary time and time spent in all different PA intensities in relation to wear time were associated with cardiorespiratory fitness and lower body muscular fitness. The relationships showed linearly increasing magnitudes with higher intensities (LPA: β = 0.17, MPA: β = 0.25, VPA: β = 0.31, MVPA: β = 0.29, total physical activity: β = 0.25, all *p* < 0.001). Similarly, positive relationships were observed between PA intensities and lower body muscular fitness, however, with weaker associations compared to VO_2max_ (LPA: β = 0.15, MPA: β = 0.19, VPA: β = 0.15, MVPA: β = 0.21, total physical activity: β = 0.19, all <0.005). Further adjustment for resistance training did not attenuate the *p*-values, although the relationships slightly attenuated. The associations of the relative distribution of time within sedentary, LPA and MVPA behaviors as a whole with cardiorespiratory and muscular fitness using compositional variables are shown in Table 3. For both cardiorespiratory and muscular fitness the proportion of time spent in sedentary and MVPA behavior were associated with cardiorespiratory and muscular fitness (SB: β = −0.63; MVPA: β = 0.79, both *p* < 0.001), while LPA was not (Table 3).

**Table 2:** Standardized regression coefficients (β) of sedentary time and physical activity (in relation to wear time) with their 95% confidence intervals (CI) for cardiorespiratory and muscular fitness adjusted for age and smoking.

	**Cardiorespiratory fitness (*****n*** **=** **405)**		**Muscular fitness (*****n*** **=** **408)**	
	**(β) (95% CI)**	***p***	**(β) (95% CI)**	***p*-value**
**Sedentary time (<1.5 MET)**				
Total sedentary time	−0.245 (−0.338; −0.152)	**<0.001**	−0.193 (−0.287; −0.099)	**<0.001**
**Mean daily accumulated sedentary time from bouts lasting**				
<3 min	0.110 (0.014; 0.206)	**0.025**	0.140 (0.045; 0.235)	**0.004**
3–10 min	−0.083 (−0.180; 0.014)	0.093	−0.045 (−0.140; 0.051)	0.358
11–19 min	−0.185 (−0.280; −0.091)	**<0.001**	−0.102 (−0.197; −0.006)	**0.036**
20–60 min	−0.152 (−0.247; −0.057)	**0.002**	−0.167 (−0.262; −0.073)	**0.001**
>60 min	−0.042 (−0.137; 0.054)	0.392	−0.117 (−0.213; −0.020)	**0.018**
**Physical activity**				
Light activity (1.6–3 MET)	0.173 (0.078; 0.269)	**<0.001**	0.148 (0.053; 0.244)	**0.002**
Moderate activity (3–6 MET)	0.246 (0.153; 0.339)	**<0.001**	0.192 (0.098; 0.286)	**<0.001**
Vigorous activity (>6 MET)	0.305 (0.212; 0.398)	**<0.001**	0.152 (0.055; 0.249)	**0.002**
Moderate-vigorous activity (≥3 MET)	0.289 (0.197; 0.381)	**<0.001**	0.212 (0.118; 0.306)	**<0.001**
Total activity (≥1.5 MET)	0.245 (0.152; 0.338)	**<0.001**	0.193 (0.099; 0.287)	**<0.001**
**Mean daily accumulated physical activity (>1.5 MET) from bouts lasting**				
<3 min	0.165 (0.069; 0.260)	**0.001**	0.165 (0.069; 0.260)	**0.001**
3–10 min	0.259 (0.166; 0.352)	**<0.001**	0.184 (0.090; 0.278)	**<0.001**
11–19 min	0.220 (0.124; 0.316)	**<0.001**	0.049 (−0.047; 0.145)	0.315
20–60 min	0.196 (0.102; 0.291)	**<0.001**	0.040 (−0.056; 0.136)	0.411
>60 min	0.113 (0.019; 0.208)	**0.019**	−0.002 (−0.097; 0.093)	0.966
**METs. maximum peak MET-level of weekly physical activity bouts lasting**				
1 min	0.281 (0.187; 0.375)	**<0.001**	0.270 (0.175; 0.365)	**<0.001**
10 min	0.312 (0.219; 0.405)	**<0.001**	0.218 (0.124; 0.313)	**<0.001**
30 min	0.333 (0.241; 0.425)	**<0.001**	0.187 (0.091; 0.282)	**<0.001**

*Bold value indicate p < 0.05*.

**Table 3:** Linear regression models estimates with *p*-values of isometric log-ratio transformed sedentary time and physical activity for cardiorespiratory fitness and muscular fitness, adjusted for age and smoking.

	**Cardiorespiratory fitness (*****n*** **=** **405)**		**Muscular fitness (*****n*** **=** **408)**	
	**Estimate**	***p***	**Estimate**	***p***
Total sedentary time vs. remaining	−0.63	**<0.001**	−0.52	**<0.001**
Light activity vs. remaining	−0.16	0.372	−0.02	0.913
Moderate-vigorous activity vs. remaining	0.79	**<0.001**	0.54	**0.003**
**Mean daily accumulated sedentary time from bouts lasting**				
<3 min vs. remaining	0.04	0.910	0.47	0.193
3–10 min vs. remaining	−0.02	0.924	−0.53	**0.040**
11–20 min vs. remaining	−0.28	0.198	0.34	0.127
>20 min vs. remaining	−0.06	0.556	−0.28	**0.009**
**Mean daily accumulated physical activity (>1.5 MET) from bouts lasting**				
<3 min vs. remaining	−0.05	0.867	−0.19	0.526
≥3 min vs. remaining	0.38	**<0.001**	0.19	**0.004**

*Remaining, remaining behaviors. Bold value indicate p < 0.05*.

In addition, relationships between accumulated PA bouts lasting less than 10 min in relation to wear time and cardiorespiratory fitness and lower body muscular fitness was observed, whereas a significant relationships between longer accumulated bout durations and only cardiorespiratory fitness were observed (Table 2). Positive relationships between Maximum peak MET-level of PA bouts and VO_2max_ and lower body muscular fitness was observed (Table 2).

The associations of the relative distribution of time within different accumulated bouts of sedentary and PA behaviors as a whole revealed that accumulated PA bouts lasting more than 3 min were associated with both cardiorespiratory (β = 0.38, *p* < 0.001) and muscular fitness (β = 0.19, *p* = 0.004). Accumulated sedentary bouts showed no associations with cardiorespiratory and muscular fitness, except that 3–10 min and >20 min bouts were associated with muscular fitness (β = −0.53, *p* = 0.04; β = −0.28, *p* = 0.009) (Table 3).

Moreover, higher cardiorespiratory fitness was related to lower volume of sedentary time and higher levels of PA intensities (*p* < 0.05 to *p* < 0.001). Similar associations were observed for lower body muscular fitness with the exception of non-significant association of LPA (Table 4).

**Table 4:** The mean (standard deviation) of the sedentary and physical activity time (min) according to physical fitness categories.

	**Cardiorespiratory**	**Fitness**			**Muscular**	**Fitness**		
	**Low (lowest 20 %)**	**Moderate (20–80%)**	**High (Highest 20%)**	***p*****-value**	**Low (lowest 20%)**	**Moderate (20–80%)**	**High (Highest 20%)**	***p*****-value**
**Sedentary time**								
Total sedentary time (min)	721 (130)	716 (133)	677 (128)	**0.042**	725 (135)	709 (132)	688 (131)	0.199
Total sedentary time (relative to wear time, %)	79.5 (8.11)	77.8 (7.76)	73.7 (7.97)	**<0.001**	79.4 (7.97)	77.2 (7.72)	75.5 (8.41)	**0.006**
**Physical activity (min)**								
Light activity (1.6–3 MET)	116 (52.1)	119 (45.0)	137 (47.7)	**0.007**	112 (47.3)	123 (46.4)	127 (46.6)	0.092
Moderate activity (3–6 MET)	66.4 (28.4)	77.8 (31.7)	94.3 (33.8)	**<0.001**	70.1 (30.5)	79.0 (32.5)	86.3 (32.6)	**0.005**
Vigorous activity (>6 MET)	2.26 (4.21)	4.06 (4.86)	7.62 (9.43)	**<0.001**	2.79 (6.03)	4.48 (6.20)	5.73 (5.62)	**<0.001**
Moderate-vigorous activity (≥3 MET)	68.7 (29.1)	81.9 (32.5)	102.0 (35.0)	**<0.001**	72.9 (31.0)	83.5 (33.9)	92.1 (33.3)	**<0.001**
Total activity (≥1.5 MET)	184 (73.4)	201 (70.5)	239 (74.1)	**<0.001**	185 (70.8)	207 (71.9)	219 (74.3)	**0.009**
**Physical Activity (relative to wear time %)**								
Light activity (1.6–3 MET)	12.8 (5.50)	13.2 (4.97)	15.1 (5.16)	**0.007**	12.4 (5.13)	13.6 (5.02)	14.2 (5.26)	0.066
Moderate activity (3–6 MET)	7.44 (3.44)	8.59 (3.50)	10.40 (3.73)	**<0.001**	7.82 (3.55)	8.72 (3.57)	9.66 (3.72)	**0.005**
Vigorous activity (>6 MET)	0.25 (0.48)	0.45 (0.53)	0.85 (1.02)	**<0.001**	0.33 (0.74)	0.49 (0.64)	0.64 (0.64)	**<0.001**
Moderate-vigorous activity (≥3 MET)	7.69 (3.53)	9.04 (3.61)	11.30 (3.82)	**<0.001**	8.14 (3.65)	9.21 (3.71)	10.30 (3.79)	**<0.001**
Total activity (≥1.5 MET)	20.5 (8.11)	22.2 (7.76)	26.3 (7.97)	**<0.001**	20.6 (7.97)	22.8 (7.72)	24.5 (8.41)	**0.006**

*Bold value indicate p < 0.05*.

### Associations of Sedentary Time and Time Spent in Different Physical Activity Intensities With Body Composition

Both sedentary time and physical activity were most consistently related to body fat percentage, whereas BMI was only related to VPA (Table 5). All PA intensities were related to rather similar magnitudes with body fat percentage (β = −0.13 to −0.16) and VPA was related to all body composition variables (*p* < 0.005). Similarly, there were relationships between the shorter accumulated PA bouts (4–10 and 11–20 min) as well as maximum peak MET-level of PA bouts and all body composition variables (*p* < 0.05) (Table 5). Moreover, some of the accumulated bouts of sedentary time were also inversely related to body fat percentage.

**Table 5:** Standardized regression coefficients (β) of sedentary time and physical activity (in relation to wear time) with their 95% confidence intervals (CI) for body mass index (BMI), waist circumference and body fat% adjusted for age and smoking.

	**BMI (*****n*** **=** **406)**		**Waist circumference (*****n*** **=** **408)**		**Body fat% (*****n*** **=** **405)**	
	**(β) (95% CI)**	***p***	**(β) (95% CI)**	***p***	**(β) (95% CI)**	***p***
**Sedentary time (<1.5 MET)**						
Total sedentary time	0.077 (−0.019; 0.182)	0.117	0.097 (0.003; 0.190)	**0.043**	0.152 (0.058; 0.247)	**0.002**
**Mean daily accumulated sedentary time from bouts lasting**						
<3 min	−0.002 (−0.100; 0.095)	0.961	−0.030 (−0.124; 0.064)	0.528	−0.131 (−0.227; −0.035)	**0.008**
3–10 min	0.051 (−0.044; 0.146)	0.293	0.078 (−0.015; 0.170)	0.100	0.078 (−0.017; 0.173)	0.109
11–19 min	0.063 (−0.033; 0.159)	0.195	0.080 (−0.013; 0.173)	0.093	0.146 (0.051; 0.241)	**0.003**
20–60 min	0.013 (−0.084; 0.110)	0.796	0.022 (−0.072; 0.115)	0.649	0.108 (0.012; 0.205)	**0.028**
>60 min	−0.013 (−0.108; 0.082)	0.790	0.006 (−0.089; 0.101)	0.901	0.033 (−0.062; 0.128)	0.492
**Physical activity**						
Light activity (1.6–3 MET)	−0.057 (−0.153; 0.039)	0.247	−0.087 (−0.181; 0.007)	0.068	−0.133 (−0.228; −0.037)	**0.006**
Moderate activity (3–6 MET)	−0.065 (−0.160; 0.031)	0.182	−0.066 (−0.160; 0.027)	0.163	−0.122 (−0.217; −0.027)	**0.012**
Vigorous activity (>6 MET)	−0.138 (−0.233; −0.042)	**0.005**	−0.139 (−0.234; −0.044)	**0.004**	−0.159 (−0.254; −0.063)	**0.001**
Moderate-vigorous activity (≥ 3 MET)	−0.086 (−0.182; 0.009)	0.076	−0.088 (−0.181; 0.006)	0.065	−0.145 (−0.240; −0.050)	**0.003**
Total activity (≥1.5 MET)	−0.077 (−0.172; 0.019)	0.117	−0.097 (−0.190; −0.003)	**0.043**	−0.152 (−0.247; −0.058)	**0.002**
**Mean daily accumulated physical activity (>1.5 MET) from bouts lasting**						
<3 min	−0.026 (−0.122; 0.071)	0.604	−0.057 (−0.152; 0.037)	0.233	−0.113 (−0.209; −0.017)	**0.022**
3–10 min	−0.144 (−0.240; −0.049)	**0.003**	−0.136 (−0.229; −0.043)	**0.004**	−0.159 (−0.255; −0.064)	**0.001**
11–19 min	−0.117 (−0.213; −0.021)	**0.017**	−0.095 (−0.188; −0.001)	**0.047**	−0.108 (−0.204; −0.012)	**0.027**
20–60 min	−0.095 (−0.190; 0.000)	0.051	−0.060 (−0.153; 0.034)	0.211	−0.083 (−0.177; 0.012)	0.088
>60 min	−0.001 (−0.095; 0.094)	0.990	0.003 (−0.090; 0.095)	0.956	0.002 (−0.093; 0.097)	0.968
**METs. maximum peak MET–level of weekly physical activity bouts lasting**						
1 min	−0.151 (−0.247; −0.055)	**0.002**	−0.156 (−0.251; −0.061)	**0.001**	−0.199 (−0.294; −0.104)	**<0.001**
10 min	−0.147 (−0.242; −0.052)	**0.003**	−0.132 (−0.226; −0.038)	**0.006**	−0.179 (−0.273; −0.084)	**<0.001**
30 min	−0.141 (−0.236; −0.045)	**0.004**	−0.124 (−0.218; −0.030)	**0.010**	−0.175 (−0.270; −0.080)	**<0.001**

*Bold value indicate p < 0.05*.

The associations of the relative distribution of time within sedentary, LPA, and MVPA behaviors as a whole with body composition using compositional variables are shown in Table 6. Accumulated PA bouts lasting more than 3 min were consistently associated with all body composition variables (β = −0.22 to −0.28, *p* < 0.001), while sedentary time was associated with body fat percentage (β = 0.42, *p* < 0.001) (Table 6). Furthermore, higher waist circumference was related to lower volume of sedentary time and greater levels of PA intensities (*p* < 0.05 to *p* < 0.001) showing little difference between overweight and obese. Conversely, only vigorous PA was higher across the BMI subgroups being highest among normal individuals with normal weight (Table 7).

**Table 6:** Linear regression models estimates with *p*-values of isometric log-ratio transformed sedentary time and physical activity for body mass index (BMI), waist circumference and body fat% adjusted for age and smoking.

	**BMI (*****n*** **=** **406)**		**Waist circumference (*****n*** **=** **408)**		**Body fat% (*****n*** **=** **405)**	
	**Estimate**	***p***	**Estimate**	***p***	**Estimate**	***p***
Total sedentary time vs. remaining	0.18	0.138	0.23	0.052	0.42	**0.001**
Light activity vs. remaining	0.02	0.924	−0.13	0.501	−0.15	0.429
Moderate-vigorous activity vs. remaining	−0.20	0.271	−0.11	0.548	−0.26	0.151
**Mean daily accumulated sedentary time from bouts lasting**						
<3 min vs. remaining	−0.44	0.223	−0.43	0.225	−0.96	**0.008**
3–10 min vs. remaining	0.15	0.569	0.33	0.198	0.44	0.091
11–20 min vs. remaining	0.09	0.688	−0.06	0.796	−0.01	0.978
>20 min vs. remaining	0.01	0.950	0.03	0.752	0.09	0.385
**Mean daily accumulated physical activity (>1.5 MET) from bouts lasting**						
<3 min vs. remaining	0.49	0.106	0.35	0.24	0.67	0.026
≥3 min vs. remaining	−0.28	**<0.001**	−0.22	**0.001**	−0.23	**<0.001**

*Remaining, remaining behaviors. Bold value indicate p < 0.05*.

**Table 7:** The distributions (%) and mean (standard deviation) of sedentary and physical activity time according to body composition categories.

		**Body mass index**				**Waist circumference**		
	**Normal weight 18.5–24.99**	**Overweight 25–29.99**	**Obese** **≥30**	***p*****-value**	**Normal weight** **<94 cm**	**Overweight 94–102 cm**	**Obese** **>102 cm**	***p*****-value**
**Sedentary time**								
Total sedentary time (min)	695 (128)	713 (134)	726 (126)	0.210	703 (136)	721 (120)	717 (131)	0.499
Total sedentary time (relative to wear time, %)	76.5 (8.39)	77.5 (7.72)	79.0 (7.29)	0.100	76.5 (8.38)	78.9 (6.33)	79.2 (7.49)	**0.016**
**Physical activity (min)**								
Light activity (1.6–3 MET)	125 (48.5)	120 (44.6)	118 (50.0)	0.496	125 (48.4)	117 (40.4)	112 (49.5)	0.133
Moderate activity (3–6 MET)	80.8 (34.6)	79.1 (30.7)	71.1 (28.4)	0.162	82.0 (34.3)	70.0 (23.6)	72.4 (30.1)	**0.006**
Vigorous activity (>6 MET)	5.08 (7.01)	4.17 (5.34)	2.34 (4.58)	**<0.001**	4.83 (6.37)	3.96 (5.76)	2.02 (3.99)	**<0.001**
Moderate-vigorous activity (≥3 MET)	85.9 (36.2)	83.3 (31.6)	73.5 (29.1)	0.063	86.8 (35.5)	74.0 (24.9)	74.4 (30.8)	**0.002**
Total Activity (≥1.5 MET)	211 (76.5)	204 (69.7)	191 (69.7)	0.205	212 (76.1)	191 (59.1)	187 (70.5)	**0.016**
**Physical Activity (relative to wear time %)**								
Light activity (1.6–3 MET)	13.9 (5.36)	13.3 (4.92)	12.8 (4.95)	0.271	13.9 (5.36)	13.3 (4.92)	12.8 (4.95)	0.117
Moderate activity (3–6 MET)	9.02 (3.86)	8.73 (3.43)	7.86 (3.33)	0.128	9.02 (3.86)	8.73 (3.43)	7.86 (3.33)	**0.007**
Vigorous activity (>6 MET)	0.57 (0.76)	0.46 (0.57)	0.27 (0.55)	**<0.001**	0.57 (0.76)	0.46 (0.57)	0.27 (0.55)	**<0.001**
Moderate-vigorous activity (≥3 MET)	9.58 (4.01)	9.19 (3.55)	8.13 (3.45)	**0.048**	9.58 (4.01)	9.19 (3.55)	8.13 (3.45)	**0.003**
Total activity (≥1.5 MET)	23.5 (8.39)	22.5 (7.72)	21.0 (7.29)	0.100	23.5 (8.39)	22.5 (7.72)	21.0 (7.29)	**0.016**

*Bold value indicate p < 0.05*.

### Joint Associations of Sedentary Time and Moderate-to-Vigorous Physical Activity With VO_2max_, Muscular Fitness and Body Composition

Individuals with high sedentary time and low MVPA had significantly lower VO_2max_ and lower body muscular fitness, and higher BMI and waist circumference (*p* < 0.05) (Table 8). There were no individuals with low sedentary time combined with low MVPA and no individuals with high sedentary time combined with high MVPA.

**Table 8:** The joint association of either low (lowest 20%) and high (highest 20%) sedentary groups combined with either low (lowest 20%) and high (highest 20%) moderate-vigorous physical activity (MVPA) groups with physical fitness and body composition (mean±standard deviation) ≠.

	**Low Sedentary time + High MVPA**	**High Sedentary time + low MVPA**	***p*-value**
Cardiorespiratory fitness (ml/kg/min)	45.4 ± 7.5	37.6 ± 8.1	**<0.001**
Muscular fitness (z-score)	0.25 ± 0.75	−0.19 ± 0.85	**0.004**
Body mass index	24.3 ± 3.0	26.0 ± 5.9	0.050
Waist circumference (cm)	84.7 ± 9.8	89.8 ± 14.5	**0.029**

*≠, There were no individuals with low sedentary time combined with low MVPA and no individuals with high sedentary time combined with high MVPA. Bold value indicate p < 0.05*.

The composition of accumulated time of the quartiles of PA intensities and sedentary time are shown in the Supplementary Figure 1.

## Discussion

The main findings of the present study revealed that both sedentary time and physical activity intensities were not only associated with cardiorespiratory fitness but also with lower body muscular fitness. Compositional analysis showed that the distribution of time spent in sedentary and MVPA behaviors, but not time spent in LPA behavior, were associated with both cardiorespiratory and lower body muscular fitness. Moreover, accumulated bout duration of physical activity lasting ≥3 min was consistently associated with cardiorespiratory fitness and lower body muscular fitness as well as all body composition indices. In compositional analysis, sedentary time but not LPA or MVPA were associated with body fat percentage.

### Cardiorespiratory Fitness

Objectively measured physical activity was positively associated with cardiorespiratory fitness in the present study similar to previous studies (Nokes, 2009; Cao et al., 2010a,b; Kulinski et al., 2014; Dyrstad et al., 2016; Gralla et al., 2016; Collings et al., 2017; Joensuu et al., 2018). It was observed that the higher the intensity of physical activity the stronger the associations between physical activity and cardiorespiratory fitness. Importantly, compositional analysis revealed that sedentary time and MVPA, but not light intensity physical activity were related to cardiorespiratory fitness showing stronger association for MVPA than sedentary time. Similarly, previous cross-sectional studies have observed that objectively measured physical activity is positively associated with cardiorespiratory fitness in adults (Kulinski et al., 2014) and children (Collings et al., 2017; Joensuu et al., 2018). Previous studies have shown that especially vigorous physical activity leads to higher maximal oxygen uptake (Weston et al., 2014). It is, however, noteworthy, that the time spent in moderate and especially vigorous physical activity in the present study were rather small, 9 % (79 min) and <1% (4 min) of the wear time, respectively. Interestingly, the amount of light, moderate and vigorous physical activity in the present study are comparable to another Finnish study in adult population aged 18–85 years (Husu et al., 2016). The small amount of particularly vigorous physical activity observed in the current study may therefore highlight its effectiveness as a time efficient strategy to increase cardiorespiratory fitness, as previously has been suggested (Gillen and Gibala, 2014). Recently, instead of planned and structured high intensity exercise training, incidental physical activity with high intensity has been suggested as a one mean to improve fitness and health (Stamatakis et al., 2019). This could be taken place during an individual‘s daily routine during active commuting or even household work (Stamatakis et al., 2019).

Interestingly, compositional analysis showed that relative time spent in light intensity physical activity was not associated with cardiorespiratory fitness within the composition of sedentary and MVPA behaviors. This finding is in line with a study in children, where light intensity PA was not associated with cardiorespiratory fitness, while MVPA and VPA were (Collings et al., 2017) Nevertheless, opposite findings have also been observed as findings from an isotemporal substitution study in adults suggest that replacing sedentary time with light intensity physical activity is associated with higher cardiorespiratory fitness (Van Der Velde et al., 2017). There is some evidence to suggest that light-intensity PA may improve selected health risk factors in adults (Chastin et al., 2019) but to the best of our knowledge, the association between light intensity physical activity and physical fitness has rarely been studied in adults. Collectively, moderate and vigorous physical activity are positively associated with cardiorespiratory fitness, whereas there are conflicting results regarding light intensity physical activity. Therefore, future studies are especially warranted to investigate the relationship of light intensity physical activity with cardiorespiratory fitness.

In the present study, we observed that also most of the accumulated physical activity bouts, whether shorter or longer, were associated with cardiorespiratory fitness. Nevertheless, the compositional analysis did not show as consistent findings as single linear regression models. PA bouts lasting more than 3 min were associated with cardiorespiratory fitness in compositional analysis. A recent review concluded that there are no differences between accumulated and continuous exercise for cardiorespiratory fitness from exercise intervention studies (Murphy et al., 2019). In addition, McGuire and Ross (2011) reported that device based measure of incidental physical activity was associated with cardiorespiratory fitness in abdominally obese men and women. The associations were stronger for intensity than the duration of incidental physical activity. These findings highlight that not only longer but also shorter bouts of physical activity may induce positive adaptations in cardiorespiratory fitness. Based on the present study findings, the higher the intensity the stronger the relationship between physical activity and cardiorespiratory fitness.

Interestingly, besides MVPA, sedentary time was also inversely associated with cardiorespiratory fitness in compositional analysis. The total time being sedentary was on average almost 12 h per day totalling of 77% of the wear time, which is very consistent with a previous Finnish study with older adult population than in the present study (Husu et al., 2016). Inconsistent findings from previous studies regarding the association between sedentary time and cardiorespiratory fitness has, however, been observed. Objectively measured sedentary time was inversely associated with cardiorespiratory fitness in adults (Kulinski et al., 2014) and in old aged individuals (Jantunen et al., 2017), whereas in children no associations between objectively measured sedentary time and cardiorespiratory fitness have been reported (Collings et al., 2017; Joensuu et al., 2018). The present study findings suggests that sedentary time is inversely associated with cardiorespiratory fitness however, the strength of that association was weaker than that of MVPA.

### Muscular Fitness

Objectively measured MVPA and sedentary time were not only associated with cardiorespiratory fitness but also with lower body muscular fitness in the present study. The associations, however, were weaker compared to those of cardiorespiratory fitness but remained significant after adjustment for resistance training. Objectively measured physical activity consists of mainly aerobic activities such as walking and running and therefore these results may partly come as a surprise. However, there are evidence that even aerobic activities and aerobic exercise can lead to increase in skeletal muscle hypertrophy (Konopka and Harber, 2014). These increases observed in the previous literature are most prominent in sedentary, inactive individuals as well as older individuals, however, similar adaptations have also been observed in young healthy adults (Konopka and Harber, 2014), such as in the present study. Furthermore, a recent systematic review (Smith et al., 2019) concluded that there was a consistent positive association of objectively measured MVPA and vigorous physical activity with strength and power in children and adolescents. Conversely, no associations of moderate or light intensity physical activity with strength and power were observed, which is in line with the present study where light intensity physical activity was not associated with lower body muscular fitness. Similarly, objectively measured vigorous physical activity, but not light or moderate physical activity, was positively associated with muscular endurance (Smith et al., 2019). Although strength training is well-elucidated to be the most effective mean to improve muscle mass and strength, it may however be speculated in light of these findings, that even aerobic physical activity may be preservative of muscular strength capabilities to some extent already in young adults, at least for when comparing to sedentary individuals. Contrary to the present study findings, Smith et al. (2019) concluded no evidence of an association between objectively measured sedentary time and strength and power, as well as inconsistent findings between sedentary time and muscular endurance.

Interestingly, the shortest analyzed physical activity bouts lasting either less than 3 or 4–10 min bouts were associated with lower body muscular fitness, whereas longer duration bouts (>10 min) were not in single regression models. In compositional analysis, PA bouts lasting more than 3 min only were associated with muscular fitness. Speculatively, it may appear that with more physical activity bouts there are more transitions from sedentary behavior to standing position and moving, which could possibly in the long term induce higher number of muscle contractions to lower extremity muscles thereby improving strength. However, evidence for this speculation should be addressed in future studies.

### Body Composition

Inverse associations were found between different physical activity intensities as well as total physical activity time and body fat percentage in single regression models, whereas the associations with BMI and waist circumference were not as consistently associated in the present study. Compositional analysis indicated that among PA variables, PA bouts lasting more than 3 min were only associated with body fat percentage. Previously, an association between anthropometric variables and objectively measured physical activity have been reported in cross-sectional studies (Besson et al., 2009; Wientzek et al., 2014; da Silva et al., 2019). However, systematic reviews conclude that in longitudinal studies there are inconsistent findings between physical activity and changes in weight and adiposity (Fogelholm and Kukkonen-Harjula, 2000; Wilks et al., 2011), although Jakicic et al. (2019) stated that there appears evidence for an inverse association between PA and weight gain from longitudinal studies. Further, intervention studies show mostly no prediction of baseline physical activity level to future change in adiposity and no effect of physical activity interventions on adiposity (Wilks et al., 2011). A recent meta-analysis concluded that high-intensity interval training reduce total, abdominal and visceral fat in individuals with overweight or obesity, however, there was no effect in normal weight. Although, high-intensity exercise has been shown to decrease total, abdominal and visceral fat, the evidence is mainly observed in overweight and obese individuals (Maillard et al., 2018) and to a less extent in normal weight individuals, such as the majority of the present study sample. Nevertheless, in the present study single regression models indicated that vigorous physical activity was associated with all of the body composition variables and the contrasting results in a review by Maillard may be explained by a very low number of studies in normal weight subjects in their review. In addition, although physical activity may be related to body composition, other obvious factors, such as nutrition intake, exist, which altogether makes the issue more complex.

Sedentary time was positively associated with body fat percentage both in single and compositional regression models. Nevertheless, similar to what has been concluded about prospective associations between physical activity and body weight, also prospective evidence of the association between sedentary behavior and body weight show inconsistent and mostly non-significant results (Campbell et al., 2018). It may appear that low physical activity and high sedentary behavior may predispose for increase in body fat content as a drivers of lower energy expenditure. In addition, sedentary behavior may be linked with increased food intake, in such sedentary behavior as watching tv. BMI on the other hand, may be less affected because it consists also of muscle mass, which can be affected by physical activity and sedentary behavior to a lesser extent. The compositional analysis showed that in the present study, accumulation of less than 3 min of sedentary time and PA bouts lasting more than 3 min were associated with body fat percentage. A recent systematic review concluded that physical activity of any bout duration are associated with health outcomes, including body composition variables (Jakicic et al., 2019). Although, controversial findings from cross-sectional studies have been observed, there are studies suggesting that there are no differences in association with BMI when comparing accumulation of <10 min bouts and >10 min bouts (Jakicic et al., 2019). Moreover, most of the review studies reported no difference in adiposity outcomes, such as visceral fat, when comparing accumulation of <10 min bouts and >10 min bouts.

### Strengths and Limitations

The strengths of the present study include not only objective measures of physical fitness and body composition but also objective measure of physical activity. The study consists of young adult men, who often are hard-to-reach focus group. Nevertheless, the present study has some limitations. Due to the cross-sectional study design, causality cannot be established, and caution should be applied when interpreting the results. As within all cross-sectional studies, a reverse causation may exist, so that for example individuals with higher body fat percentage are less likely to engage in physical activity than vice versa. The assessment of body composition was based on bioimpedance method, known to be biased to some limitations. The equation used assumes that total body water is a constant fraction of fat free mass but many conditions can affect this within a group and individual level. Although, our procedures were standardized before the measurements this limitation has to be taken into account. Secondly, although a relatively good agreement between single and multi-frequency methods has been reported against total body water by dilution, a variance of error is large and can be over 2 l (~5%) for total body water producing high inter-individual variation. Bioimpedance method has therefore been reported to give systematically lower body fat and higher body lean mass values (Sillanpää et al., 2014).

## Conclusions

In conclusion, the present study revealed that both sedentary time and moderate-to-vigorous physical activity were associated with cardiorespiratory fitness and also with lower body muscular fitness in young healthy adult men. Moreover, accumulated bout duration of physical activity lasting ≥3 min was consistently associated with cardiorespiratory fitness and lower body muscular fitness as well as all body composition indices. The present findings emphasize the importance of avoiding sedentary time and adopting physical activity at moderate-to-vigorous intensity levels, which may further have beneficial associations on physical fitness and body composition and mediate health effects.

## Data Availability Statement

Data are available on reasonable request. The Finnish Defence Forces own and manage the data, which are available for researchers who meet the criteria for access to confidential data.

## Ethics Statement

The studies involving human participants were reviewed and approved by ethical committees of the University of Jyväskylä and the Central Finland Health Care District, as well as the Defence Command of the Finnish Defence Forces (AM5527). The patients/participants provided their written informed consent to participate in this study.

## Author Contributions

JV participated in the design of the study, contributed to data collection and data analysis and interpretation of results. TV participated in the design of the study, contributed to data analysis and interpretation of results. TW, KP, and TO participated in the design of the study and contributed to interpretation of results. JR and HV-Y contributed to data analysis and interpretation of results. HK participated in the design of the study, contributed to data collection and interpretation of results. All authors have read and approved the final version of the manuscript, and agree with the order of presentation of the authors.

## Conflict of Interest

The authors declare that the research was conducted in the absence of any commercial or financial relationships that could be construed as a potential conflict of interest.

## Supplementary Material

Supplementary Figure 1 Compositional analysis of the relative importance of the group mean time spent in sedentary, LPA and MVPA in regards to the overall mean time composition by groups of fitness and body composition outcomes.
