# Supplementary figures and images for: Device-Based Measures of Sedentary Time and Physical Activity Are Associated With Physical Fitness and Body Fat Content
Source: Front Sports Act Living. 2020 Dec 18;2:587789. doi: 10.3389/fspor.2020.587789 (PMC7750877; doi:10.3389/fspor.2020.587789)

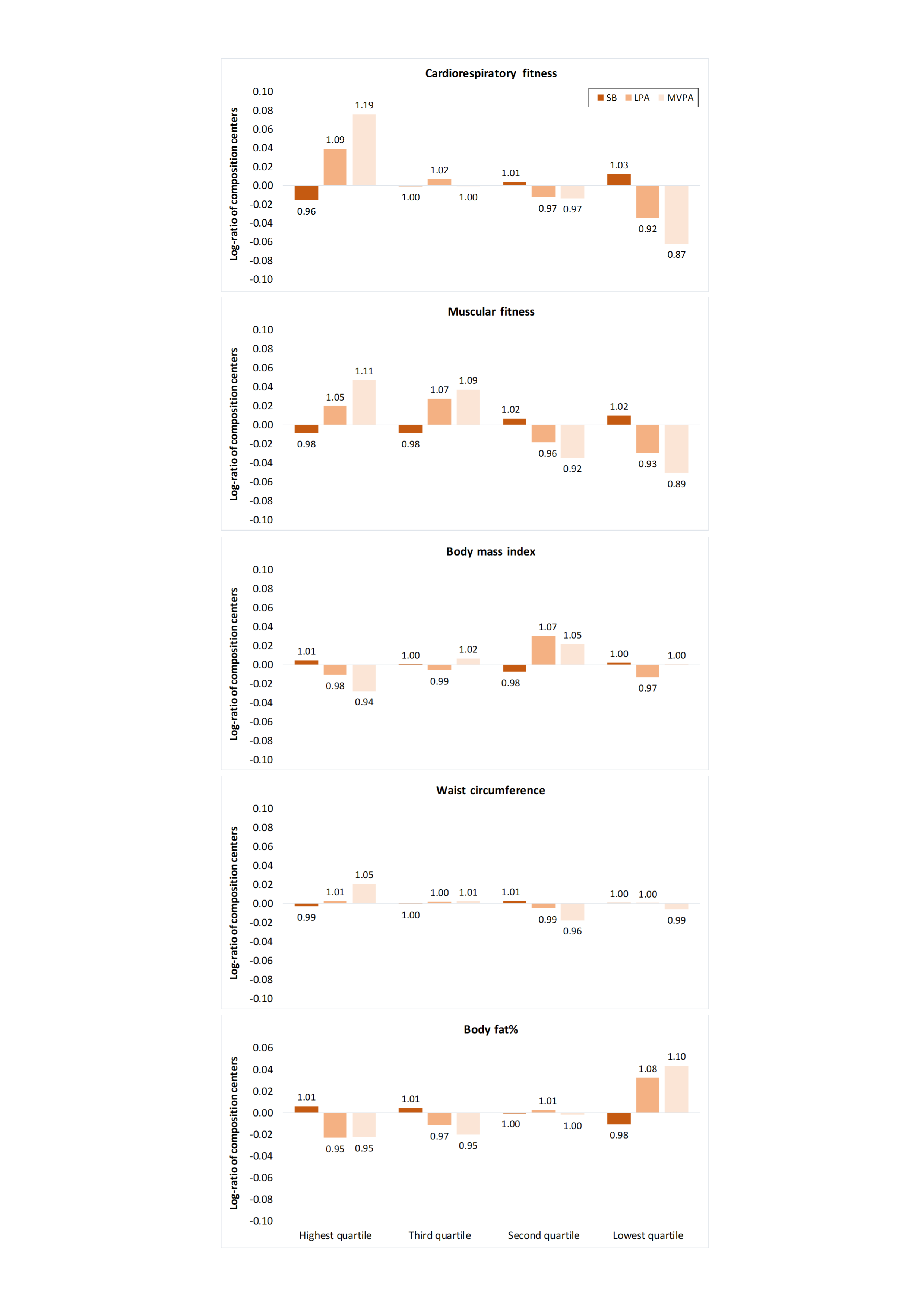

Supplement: Supplementary Figure 1 — Compositional analysis of the relative importance of the group mean time spent in sedentary, LPA and MVPA in regards to the overall mean time composition by groups of fitness and body composition outcomes. [file Image_1.TIF]
